# Antidepressant-like Effects of *Polygonum minus* Aqueous Extract in Chronic Ultra-Mild Stress-Induced Depressive Mice Model

**DOI:** 10.3390/bs12060196

**Published:** 2022-06-18

**Authors:** Muhammad Irfan Bashir, Nur Hidayah Kaz Abdul Aziz, Dzul Azri Mohamed Noor

**Affiliations:** 1 Discipline of Pharmacology, School of Pharmaceutical Sciences, Universiti Sains Malaysia, Minden 11800, Penang, Malaysia; 2 Discipline of Clinical Pharmacy, School of Pharmaceutical Sciences, Universiti Sains Malaysia, Minden 11800, Penang, Malaysia; dzulazri@usm.my

**Keywords:** anti-depressant, *Polygonum minus*, aqueous, chronic, ultra-mild, stress

## Abstract

Depression is the most common behavior disorder that leads to many disabilities. The main aim of this study was to evaluate the effects of a *Polygonum minus (P. minus)* aqueous extract on chronic ultra-mild stress (CUMS)-induced depressive mice model. Chronic ultra-mild stress can disturb the neurotransmitters levels and plasticity of the hippocampus. Balb/c male mice were used in this study, which consisted of six groups (*n* = 14). Treatment was given for eight weeks, and chronic ultra-mild stress was applied for six weeks. Commercially available *P. minus* extract (BioKesum^®^) was used in this study. The behavior and neurochemical parameters were investigated through behavioral Tests and ELISA assays. *P. minus* administration significantly *(p <* 0.05) restored CUMS-induced behavior abnormalities, decreased the immobility time, and increased the sucrose preference and increased the spatial memory. *P. minus* treatment also showed the decreased level of serum corticosterone and increased the level of hippocampal neurotransmitters (Serotonin and Norepinephrine) significantly *(p <* 0.05). The brain-derived neurotrophic factor (BDNF) level also increased significantly in both the prefrontal cortex and hippocampus *(p <* 0.05). *P. minus* treatment exhibited significant (*p <* 0.05) reduction of Monoamine Oxidase-A (MAO-A) in the hippocampus. These findings indicate that *P. minus* aqueous extract exhibits antidepressant effects, including decreased immobility time, increased spatial memory, reduced corticosterone, increased BDNF level, and reduced MAO-A enzyme level with increasing the monoamines (serotonin and norepinephrine) in the hippocampus.

## 1. Introduction

Depression is a common illness around the world. According to a WHO report, around 350 million people of all ages are influenced by this disorder. It is a heterogeneous syndrome and can lead to poorer quality of life. In the worst case, depression can also lead to suicide [1,2]. Several clinical features have been linked to the severity levels of mood disorders including cognitive impairment, anxiety, insomnia, and hopelessness [3,4]. Depressive disorders are expensive and are associated with significant morbidity and mortality as well [5]. There are some hypotheses related to the pathophysiology of depression, such as the neurotrophic hypothesis (reduction of a neurotrophic factor in the brain, imbalance of monoaminergic systems (imbalance of serotonergic, noradrenergic and dopaminergic neurotransmission), and HPA axis dysfunction (hypercortisolemia) hypothesis [6,7]. Clinical evidence also suggested that disorders of serotonin (5-HT) [8], norepinephrine (NE), and dopaminergic neurotransmission in the central nervous system are the main causes of depression. Almost all currently available antidepressants act on one or more of the stated mechanisms, such as the inhibition of 5-HT or NE reuptake, antagonism of inhibitory presynaptic 5-HT or NE receptors, or the inhibition of monoamine oxidase. All these mechanisms lead to an increase in the concentration of 5-HT and NE in the brain [9]. Another target for treating depression is a monoamine oxidase (MAO), an enzyme responsible for breaking down monoamines [10]. Many researchers concluded that flavonoids, alkaloids, coumarins, and their derivatives from natural herbal medicines have a strong MAO-A inhibition ability. These natural drugs became suitable choices for screening MAO-A inhibitors, as they show a synergistic pattern with antidepressant-like effects. A large variety of plant extracts and their isolated components with MAO-A inhibitory activity are now used for treating depression [11,12]. New antidepressant agents are needed that are inexpensive, rapid-acting, effective, and safe [13].

Stress is a common reason for depression. Chronic stress causes major depressive disorders by altering neurotrophic and neuroinflammatory factors in an organism [14]. The chronic ultra-mild Stress (CUMS) is an authentic, validated, and widely used animal model of depression that causes the alteration of animal behavior and nervous system and consists of a different schedule of minor stress protocols [15,16]. Recent studies have reported that the chronic ultra-mild stress (CUMS) procedure—which is the prolonged recurrence of low-intensity stressors, including social isolation, circadian rhythm disturbances, and changes in daily living habits—induces mood disorders in mice [17]. Neurobiological abnormalities in mice treated with CUMS would be similar to those observed in patients with depression, such as decreased BDNF in the hippocampus or the suppression of neurogenesis. The hippocampus is one of the limbic structures associated with emotional responses and is the most studied brain region in depression research. From the point of view of neuronal plasticity, it is generally accepted that hippocampal plasticity plays an important role in the onset and development of depression. Therefore, measuring neurotransmitters in the hippocampus that target the brain is very useful for studying the mechanisms underlying disease processes and treatment goals for chronic ultra-mild depressive stress [17,18].

*Polygonum minus* Huds, the synonym for Persicaria minor comes from the Polygonaceae family and is commonly known as Kesum or Laksa leaf in Malaysia. It is used as a flavoring in many dishes and is also consumed as ulam (salad) for health care [19]. The *P. minus* leaf extract contained flavonoids such as quercitrin (quercetin 3-rhamnoside) and quercetin 3. glucuronide has been suggested to have antioxidant, anti-inflammatory, antimicrobial, and cognitive-enhancing properties. An aqueous extract *P. minus* showed significant enhancing effects on memory and cognitive functions with a 100 mg/kg dose in mic [20] e. Recent research using *P. minus* extract has shown significant improvements in attention and memory, mood, and quality of life among middle-aged women after six weeks of administration. BioKesum is a Malaysian brand of *P. minus* leaf extract which consist of standardized flavonoids including quercitrin and quercetin 3. glucuronide. The latest research of six months of BioKesum^®^ administration potentially improved visual memory, and negative mood among older patients but its mechanism was unknown. BioKesum^®^ was developed and registered under the National Pharmaceutical Control Bureau (NPCB) with the registration number of MAL14015033T [21]. The anti-depressant potential of this plant regarding its effects on MAO-A enzyme, BDNF, neurotransmitters (serotonin and norepinephrine), and stress hormones such as corticosterone, were undiscovered, which is why this plant has been selected for further research in this study. 

The main aim of this study was to evaluate the effects of *Polygonum minus* aqueous extract on stress-induced depressive, and anxiety behavior in mice through measurement of various neurochemicals.

The current study investigated the effects of *Polygonum minus* on chronic ultra-mild stress-induced mice models through various behavioral and experimental assays. Behavioral tests consisted of the sucrose preference test (SPT), Barnes Maze assay, open field test (OFT), and forced swimming test (FST). ELISA assays were performed for the measurement of the brain-derived neurotrophic factor (BDNF), serotonin (5HT), norepinephrine (NE), monoamine oxidase A (MAO-A, and serum corticosterone level.

Male Balb/c mice (*n* = 14) were used in this study because the latest research about the cognitive effect of this extract was on male mice [20]. The study consisted of six groups. The control group was treated with simple distilled water, and the negative control was a simple CUMS group (the only stress group without treatment, though distilled water was administered); the positive control received amitriptyline at 20 mg with CUMS, and there were three treatment groups based on different doses (*P. minus* 50 mg,100 mg, and 200 mg with CUMS). Treatment was continued for eight weeks and chronic ultra-mild stress was applied for six weeks. The chronic ultra-mild stress protocol included some interventions like restraining stress, food, and water deprivation, wet bedding, cage tilting, tail pinching, foreign object incorporation, forced swimming, crowded housing, and continuous lighting. 

## 2. Materials and Methods

### 2.1. Chemicals and Drugs

BioKesum^®^, a water-soluble *Polygonum minus* aqueous extract containing flavonoids extract from its leaf part, as commercially available, was obtained from Biotropics Malaysia Berhad. Amitriptyline (Apotex Inc, Toronto, ON, Canada), serotonin, norepinephrine, monoamine oxidase A, serum corticosterone, and brain-derived neurotrophic factor (BDNF) Elisa kits were purchased from (Qayee Bio-Technology Co., Ltd. Unit 201–229 Shanghai, China). 70% Ethanol, Sucrose, and Phosphate buffer (Sigma-Aldrich, Inc, Taufkirchen, Germany).

### 2.2. Animals and Experimental Protocol

Adult male balb/c mice (22–26 g) were purchased from the Animal Research and Service Centre, Kelantan Campus, Universiti Sains Malaysia. All experimental procedures were carried out after getting the approval from USM Institutional Animal Care and Use Committee (USM IACUC) of Universiti Sains Malaysia (No of Animal Ethical Approvals: USM/IACUC/2020/(123)(1075) & USM/IACUC/2021/ (127) (1129).

The animals were housed under standard environmental conditions (24 ± 1 °C) in the Animal Transit Room at the School of Pharmaceutical Sciences, Universiti Sains, Malaysia. They were maintained on a 12-h light/dark cycle. The animals were randomly separated into six experimental groups. A chronic ultra-mild stress method was adopted to induce stress in the animals. The normal control group was without any chronic ultra- mild stress (CUMS), and the remaining groups were influenced by chronic ultra-mild stress (CUMS). Chronic ultra-mild stress remained for six weeks. Treatment remained for eight weeks means until the end of behavioral tests and sampling. 

**Figure 1:**
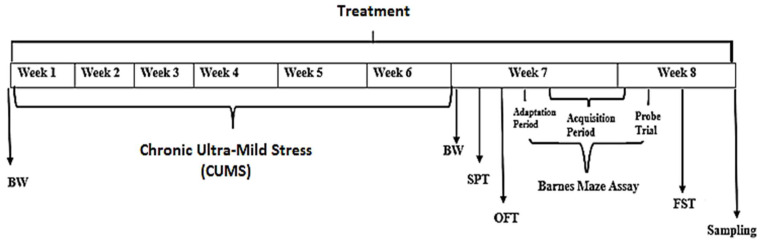
Schematic experimental design. BW means measurement of body weight, SPT means Sucrose preference test, OFT means Open field test and FST stands for Forced swimming test.

Group 1: Control group (Without CUMS) treated with distilled water only (*n* = 14). Group 2: (CUMS group, a negative Control) Chronic ultra- mild stress (CUMS) treated with 6 weeks of CUMS and distilled water for 8 weeks (*n* = 14). Group 3: CUMS + Amitriptyline 20 mg, a Positive Control (*n* = 14). Group 4: CUMS + *P. minus* treated group with 50 mg/kg (*n* = 14). Group 5: CUMS + *P. minus* treated group with 100 mg/kg (*n* = 14). Group 6: CUMS + *P. minus* treated group with 200 mg/kg (*n* = 14). Treatment was given through the oral route by using Oral gavage for mice. A complete design of the study is expressed in Figure 1.

### 2.3. Chronic Ultra Mild Stress Protocol

Chronic Ultra mild stress was given for 6 weeks. A weekly stress plan was made as shown in Table 1. Two different stressors were given on daily basis, first stress was applied in the morning and the second stress in the evening as followed by the latest research [16,22].

### 2.4. Body Weight Test

The body weight of each animal was measured at 0 day and after 6 weeks.

### 2.5. Behavioral Tests

#### Sucrose Preference Test

Mice were exposed to both the test solution (1% sucrose) and tap water for a 24 h period before 2 days from the experimental trial. Mice were food and water-deprived for 14 h before the start of the experiment. During the test, sucrose preference was evaluated for 6 h by using two bottles of 1% sucrose and tap water and the position of the bottles was switched after 3 h. The sucrose preference was calculated by the ratio of the consumed sucrose solution to the total amount of liquid consumed [23].

### 2.6. Open Field Test

The open-field test is a classic and well-validated test for detecting the anxiety and locomotion activity of animals. Many researchers explained that an anxious object shows less movement in the central zone as compared to normal, and they spend more time in the periphery and not in the central zone. Thigmotaxis theory and rearing can also provide an idea as an exploratory measure for anxiety [24,25]. An automated open-field apparatus (Pan Lab, Spain) was used to detect the parameters as distance travelled in the central zone, total distance travelled, time spent in the central zone, and the number of rearing. The apparatus had a Perspex cage (height: 40 cm, length: 90 cm; width: 90 cm), with the five zones divided into the bottom. The detection unit consisted of a 45 × 45 cm frame containing a total of 16 × 16 infrared beams at an interval of 2.5 cm located on the sides, and there were 32 cells in one frame. The mice’s movements were stored and analyzed. For testing, each animal was placed in the center of the open field and spontaneous activity (travelling and rearing) was recorded for 10 min (*n* = 14/group) [26].

### 2.7. Barnes Maze Assay

The Barnes Maze procedure was first developed by Carol Barnes. It is a behavioral test based on dry-land. It was developed to study spatial memory in rodents [27]. The Barnes Maze consisted of a wooden circular platform with 12 holes placed 2.5 cm from the edge, and all holes were equally distributed around the surface. Its surface platform was 92 cm in diameter and 92 cm above the ground. The Barnes Maze takes advantage of the animal’s natural reluctance to open up lit places. Therefore, subjects are motivated by a bright light to locate an escape hole that leads to a dark box. The Barnes Maze test consisted of three phases: an adaptation period, an acquisition period, and a probe trial. Before starting the test on the first day, a preliminary test (adaptation period) was performed. In this study, the acquisition trial consisted of 5 days and then a probe trial was performed twenty-four hours after the final acquisition trial. The behavior of the experimental subject was captured by a video camera and was recorded on the computer. Four parameters were observed in this assay: the total path length, number of errors (number of wrong holes explored during acquisition trail), latency (the time taken to escape from target hole during acquisition trial days), and the percentage of time spent during the probe trial [23]. Kinovea^®^ (0.8.15) a video tracking software was used to track the path length.

#### 2.7.1. Adaptation Period

During this period, the subject was placed in the center area on the maze surface. A chamber was placed on mice for 10 s after it was removed, and the subject was allowed to explore the maze for 30 s, then was gently guided towards the escape hole. If the subject did not enter the escape hole, it was placed inside the black box. The hole was then covered and the mice remained there for 3 min. After this, the subject was returned to its original cage and the surface of the maze was cleaned with 70% ethanol.

#### 2.7.2. Acquisition Period

In this period, the subject could explore the maze for 8 min and then be gently guided to the escape hole. If the subject did not enter the escape hole, it was placed inside. The hole was then covered and the subject remained there for 1 min. The subject was returned to its home cage and the platform was cleaned with 70% ethanol. All movements of mice were recorded by the camera every day.

#### 2.7.3. Probe Trial

In the probe trial, the subject was placed in the center zone of the maze surrounded by a chamber for 10 s. The chamber was removed and the subject was fully allowed to explore the area for sharp 90 s after the subject was returned and the surface was cleaned with 70% alcohol [28].

#### 2.7.4. Forced Swimming Test

Mice were introduced into a glass cylinder (50 cm deep, 30 cm in diameter) filled with water up to a height of 40 cm from the base and forced to swim for 6-min. The swim session was recorded by a video recorder. After each swimming session, the mice were removed from the cylinder, dried with towels, and placed in a temporary resting cage for 20 min, and then mice were returned to their home cage. Water in the cylinder was renewed after every 6 subjects. The time of swimming and immobility was recorded using a stopwatch. The duration of each behavior was calculated [29].

### 2.8. Experimental Assays

#### Blood Sample Collection

The blood samples were collected by cardiac puncture and were kept at room temperature for 30 min, then centrifuged at 3000 rpm for 20 min for serum collection, and stored at −80 °C for the corticosterone assays [11].

### 2.9. Brain Tissue Sample Collection and Homogenization

The mice were sacrificed, and the brains were quickly removed on ice. The prefrontal cortex and hippocampus were isolated and stored at −80 °C. Further at the time of assays, the prefrontal cortex and hippocampus were weighed and homogenized by 1:4 *w*/*v* with phosphate buffer saline (PBS- Sigma 7.2–7.6). The supernatant was collected for further assays after centrifuging it at 12,000 rpm [11].

### 2.10. Serum Corticosterone Assay

Serum corticosterone level was measured by using an enzyme-linked immunosorbent assay (ELISA) kit (Qayee Bio-Technology Co., Ltd. Unit 201–229 Shanghai, China) by following the manufacturer’s protocol at 450 nm absorbance in a microplate reader.

### 2.11. Measurement of Brain-Derived Neurotrophic Factor (BDNF) Level

The brain-derived neurotrophic factor BDNF level was measured from both hippocampus and prefrontal cortex separately by using an enzyme-linked immunosorbent assay kit (ELISA) kit (Qayee Bio-Technology Co., Ltd. Unit 201–229 Shanghai, China) by following the manufacturer’s protocol at 450 nm absorbance in a microplate reader.

### 2.12. Measurement of Serotonin (5HT) and Norepinephrine (NE), Monoamine Oxidase A (MAO-A) from the Hippocampus

The concentrations of serotonin, norepinephrine, and monoamine oxidase A (MAO-A) were measured from the hippocampus only by Elisa Kits (Qayee Bio-Technology Co., Ltd. Unit 201–229 Shanghai, China) at 450 nm by using a microplate reader and all protocol was followed absolutely as provided by the manufacturer.

### 2.13. Statistical Analysis

Statistical analysis was performed using SPSS version 26 statistical software (IBM, New York, NY, USA). Most data were analyzed by one-way ANOVA followed by post hoc comparisons between all groups by using Tukey HSD, except for the Barnes Maze assay. For the Barnes Maze assay, data of three parameters (travelled distance, latency, and the number of errors) were analyzed by repeated measure ANOVA. Differences in mean values with *p <* 0.05 were considered as a level of significance in all tests. The means were calculated with standard error means (SEM).

## 3. Results

### 3.1. Effects of P. minus Aqueous Extract on Body Weight 

**Figure 2:**
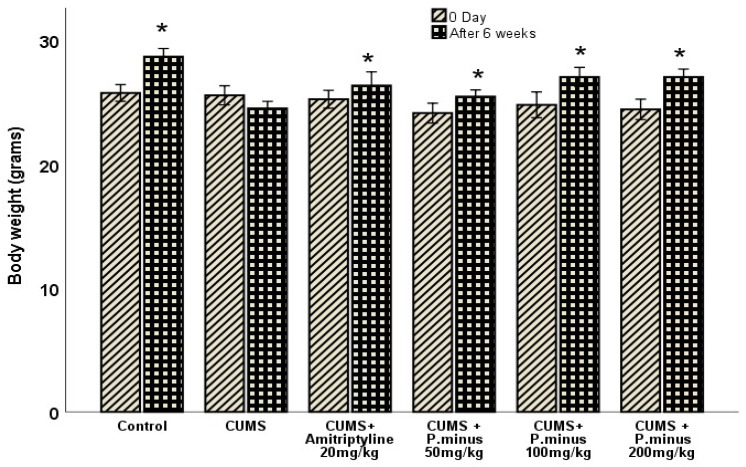
Effects of *P. minus* aqueous extract on body weight of CUMS induced stressed mice. * (*p <* 0.05), vs. zero-day of each group, (*n* = 14).

Figure 2 shows the relationship between the body weight of mice at zero-day and after 6 weeks. The body weight results showed an increasing trend in all treatment groups significantly in comparison to their zero-day measurements, except for the CUMS group. The CUMS group showed a non-significant difference in body weight as compared to its zero-day value. 

### 3.2. Behavioral Tests Results

Effects of *P. Minus* Aqueous Extract on Sucrose Preference Test.

**Figure 3:**
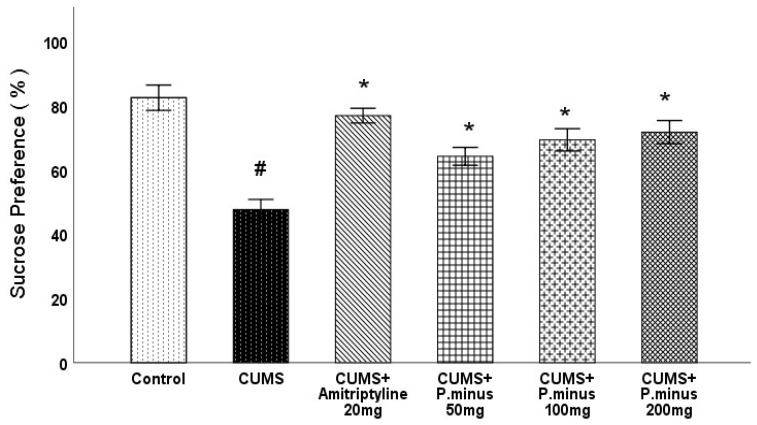
Effects of *P. minus* aqueous extract on sucrose preference test in CUMS induced depressive mice. # (*p <* 0.05), vs. Control group; * (*p <* 0.05), vs. CUMS group, (*n* = 14).

As depicted in Figure 3, the CUMS group showed a significant (*p <* 0.05) decrease in sucrose preference in comparison to the control group, which was improved in all treatment groups significantly compared to CUMS group. CUMS+ Amitriptyline 20 mg showed larger, and CUMS + *P. minus* 50 mg showed minimum sucrose preference among all treated groups.

### 3.3. Effects of P. minus Aqueous Extract on Open Field Test

**Figure 4:**
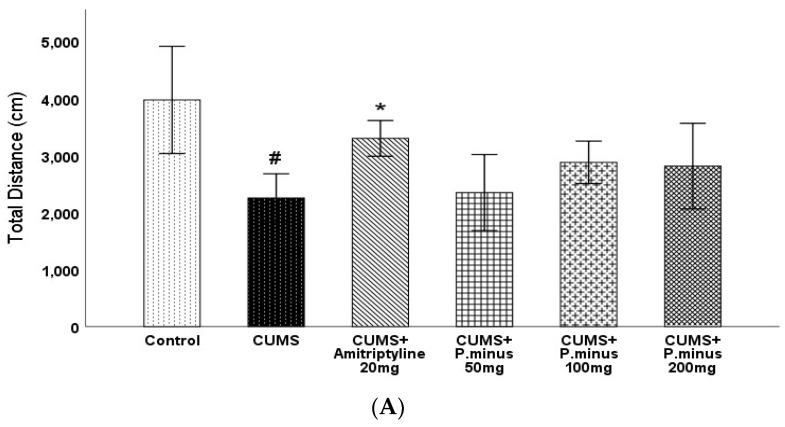

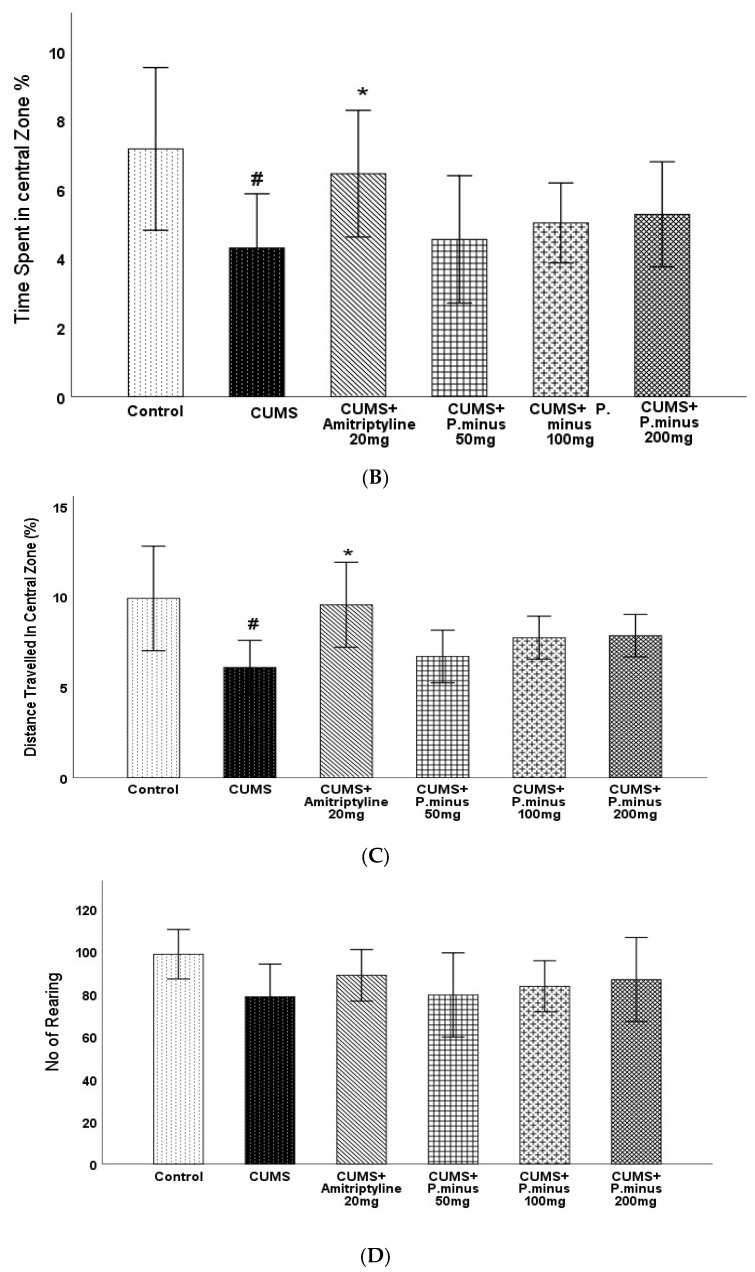
Effects of *P. minus* aqueous extract on Open Field Test in CUMS induced depressive mice. (**A**) Total distance travelled (cm), (**B**) Time spent in central zone (%), (**C**) Distance travelled in central zone. (**D**). No of rearing # (*p <* 0.05), vs. Control group; * (*p <* 0.05), vs. CUMS group. (*n* = 14).

Open field test results have been expressed in four parts: total distance travelled by mice (Figure 4A), percentage of time spent in the central zone (Figure 4B), distance travelled in the central zone (Figure 4C), and no of rearing (Figure 4D). In the first part (A), the CUMS group showed a less travelled distance with a *p <* 0.05 level of significance as compared to the control, which was only reversed by CUMS + Amitriptyline 20 mg group by *p <* 0.05 significance. The remaining CUMS+ *P. minus* treated groups showed a non-significant increase in travelled distance as compared to CUMS group. For the percentage of time spent and covered distance in the central zone, results have been shown in Figure 4B,C respectively, which expressed that the CUMS group had shown a significantly (*p <* 0.05) small residence time and shorter distance in the central zone in comparison to the control group. Only CUMS + Amitriptyline 20 mg treatment improved timings and travelled distance in the central zone significantly (*p <* 0.05), whereas the CUMS + *P. minus* 50 mg,100 mg and 200 mg groups showed non-significance improvement in both timings and distance as compared to CUMS group. Results for rearing behavior are presented in Figure 4(D), which indicates that there was no significant difference between the control and CUMS groups, as well as all remaining treated groups had non-significant differences in comparison to the CUMS group.

### 3.4. Effects of P. minus Aqueous Extract on Barnes Maze Assay

**Figure 5:**
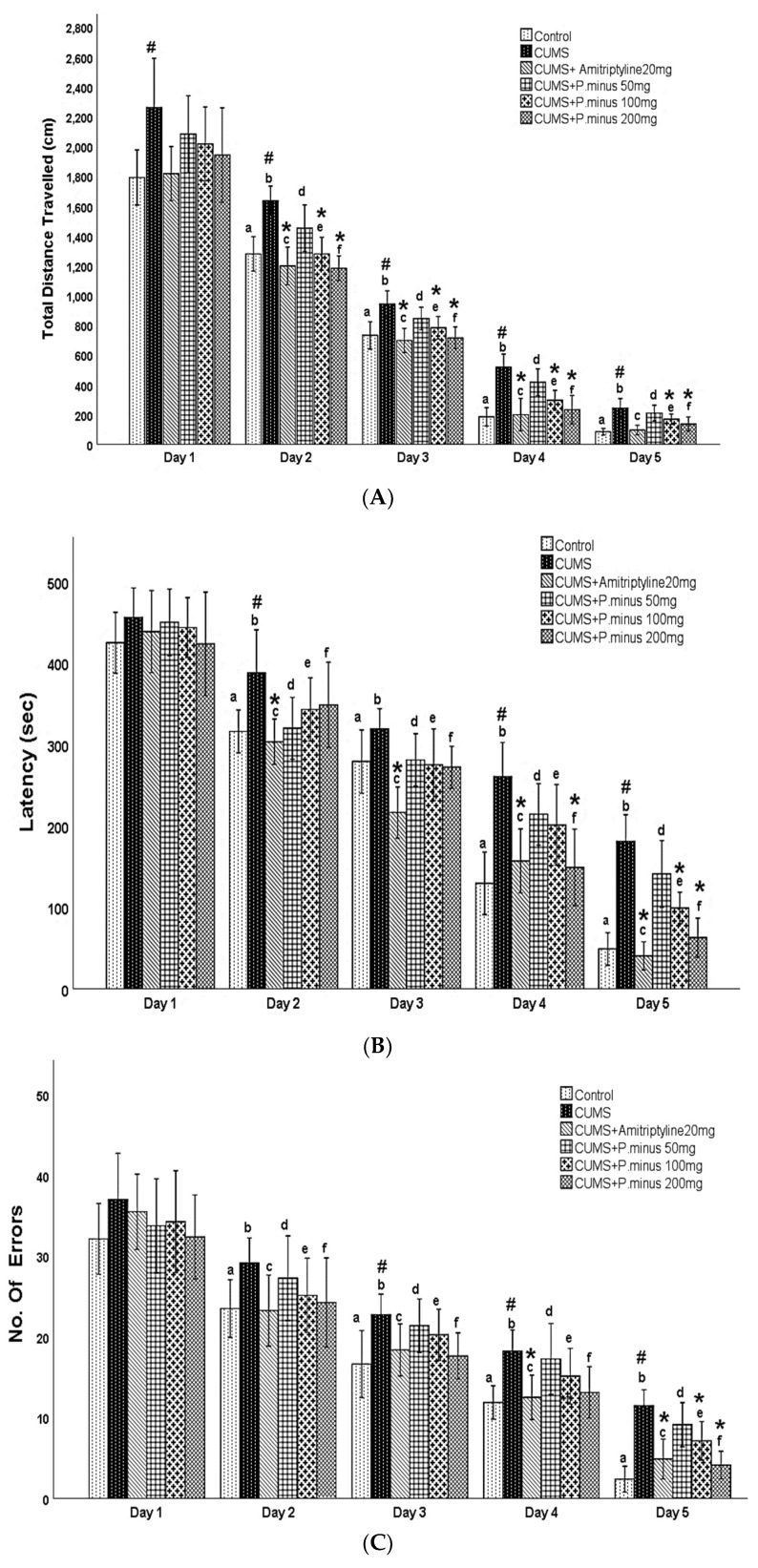

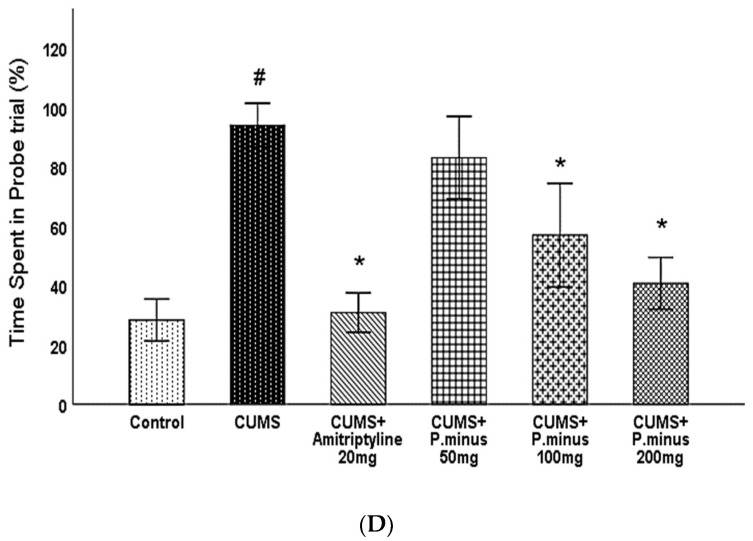
Effects of *P. minus* aqueous extract on Barnes Maze Assay in CUMS induced depressive mice. (**A**)Total distance travelled (cm), (**B**) Latency (sec), (**C**) No of Errors (**D**) Time spent during prob trial **#** (*p <* 0.05), vs. Control group; * (*p <* 0.05), vs. CUMS group with respect to each day. CUMS group. **a** showed significant variance between the Control group (Day 1) and control groups from other days, **b** showed significant variance between CUMS group (Day 1) and other CUMS groups. **c** showed significant variance between CUMS + Amitriptyline20 mg group (Day 1) with other CUMS + Amitriptyline 20 mg groups on different days. **d** showed significant variance in between *P. minus* 50 mg group (Day 1) with other *P. minus* 50 mg groups on different days. **e** showed significant variance between *P. minus* 100 mg group (Day 1) with other *P. minus* 100 mg groups on different days, whereas **f** showed significant variance between *P. minus* 200 mg group (Day 1) with other *P. minus* 200 mg groups on different days. Whereas *n* = 14.

The results of the Barnes maze are expressed in Figure 5. As shown in (A) CUMS group had travelled a larger distance as compared to the Control group significantly, which showed that the control group had completed its task by covering less distance in comparison to CUMS group. On day 1, there was no significant difference between CUMS group and treatment groups, but on Day 2, significant differences appeared. From Day 2 to Day 4 all treatment groups (CUMS + Amitryptyline20 mg, CUMS + *P. minus* 100 mg, CUMS+ *P. minus* 200 mg) except CUMS + *P. minus* 50 mg showed a significant less travelled distance as compare to CUMS. On the last day of the acquisition trial (Day 5), only CUMS + Amitriptyline 20 mg and CUMS+ *P. minus* 200 mg showed a significant decrease in the travelled distance in comparison to CUMS group. Part B of Figure 5 presented the results for latency time (the taken by subjects to escape from the hole). According to the results, CUMS group showed a significantly higher latency period as compared to the control in the last two days (Day 4 and Day 5), whereas, on Day 2, this difference was also significant. Day 1 results showed no significant difference between any groups, from day 2 to day 5 CUMS + Amitriptyline 20 mg group showed significantly less latency time in comparison to the simple CUMS group. It was observed that among *P. minus* treatment groups CUMS + *P. minus* 100 mg and CUMS + *P. minus* 200 mg showed significant less latency in comparison to CUMS group. The same pattern had been observed in CUMS+ *P. minus* 200 mg for the last 2 days (Day 4 and Day 5), whereas CUMS + *P. minus* 100 mg expressed this result only on the last day (Day 5). Figure 5 presents results for the number of errors during the acquisition trial in part C. Results related to this part showed that CUMS group had made larger numbers of errors on all days during the acquisition trial, but a significant difference was reported on Day3 and Day 4 by *p <* 0.05, whereas Day 5 was also significant. In the case of comparison between the treatment group and CUMS group, there was no significant difference in these groups from Day 1 to Day 3, at Day 4 CUMS + Amitriptyline showed a significantly small number of errors by *p <* 0.05, and on Day 5 this group also showed significance variance with decreased number of errors. Among *P. minus* treated groups, CUMS + *P. minus* 100 mg showed a significantly small number of errors on Day 5 with *p <* 0.05, and CUMS + *P. minus* 200 mg group also showed a significance in the decreased number of errors on Day 5. The results of the probe trial in form of percentage time spent by subjects to achieve their task in fixed provided time have been shown in the D part of Figure 5, these results expressed that CUMS group had spent significant (*p <* 0.05) time to reach their destination in comparison of Control group. CUMS + Amitriptyline 20 mg, CUMS + *P. minus* 100 mg, and CUMS + *P. minus* 200 mg groups have been showing a significantly shorter time to escape from a hole than the CUMS group.

As a whole, the results of Barnes maze analysis showed that all groups learnt about the task day by day, but CUMS group and CUMS + *P. minus* 50 mg group were significantly slower than other groups, whereas CUMS + Amitriptyline, CUMS + *P. minus* 200 mg and CUMS + *P. minus* 100 mg showed significantly better power of learning and efficient memory characteristics than CUMS group.

### 3.5. Effects of P. minus Aqueous Extract on Forced Swimming Test (FST)

**Figure 6:**
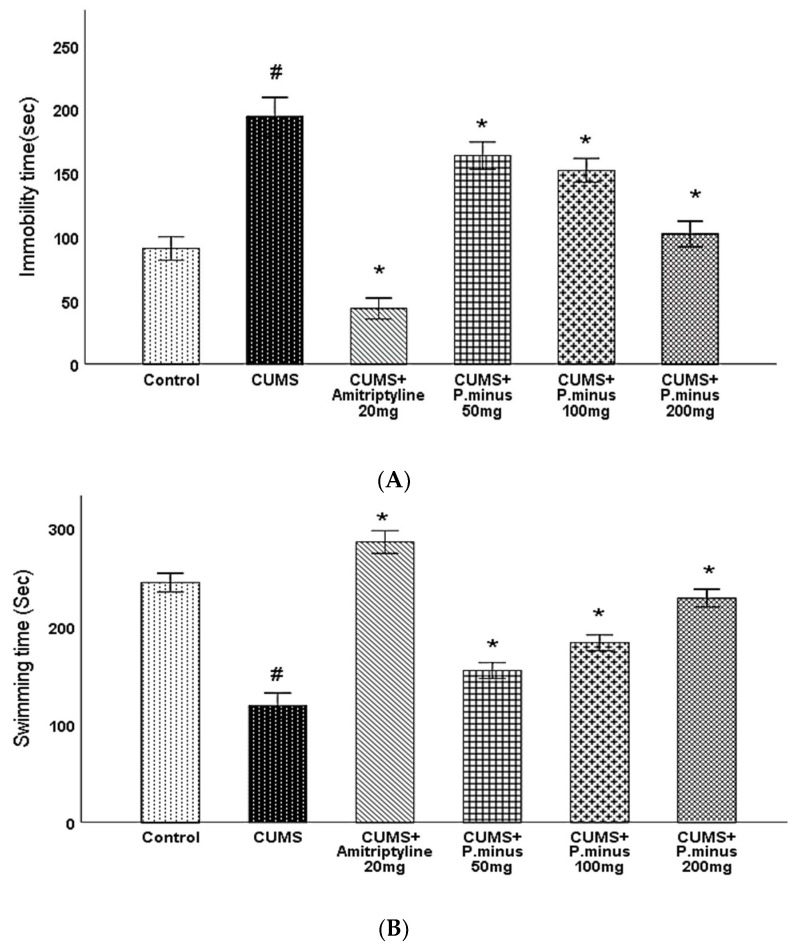
Effects of *P. minus* aqueous extract on Forced Swimming Test (FST) in CUMS induced depressive mice. (**A**) Immobility time(sec) (**B**) Swimming time(sec). **#** (*p <* 0.05), vs. Control group; * (*p <* 0.05), vs. CUMS group. (*n* = 14).

As sketched in Figure 6A, CUMS group showed an increased immobility time which indicates that CUMS had induced depression significantly which was reversed in all treatment groups. CUMS + Amitriptyline 20 mg and CUMS + *P. minus* 200 mg reduced immobility by *p <* 0.05 level of significance, whereas CUMS + *P. minus* 100 mg and 50 mg treated groups also reduced immobility significantly. The results of swimming behavior during the forced swimming test are presented in (B) part of Figure 6, which expressed that swimming time was significantly less in CUMS group as compared to the Control. This reduced swimming time was significantly enhanced in CUMS + Amitriptyline 20 mg, CUMS + *P. minus* 200 mg and *P. minus* 100 mg. The significance level of improved swimming behavior in CUMS + *P. minus* 50 mg treated group was *p <* 0.05. The results demonstrate the anti-depressant effect of Amitriptyline and *P. minus* treated groups with CUMS induced depression model of mice.

### 3.6. The Results of Experimental Assays 

#### 3.6.1. Effects of *P. minus* Aqueous Extract on Serum Corticosterone Level

**Figure 7:**
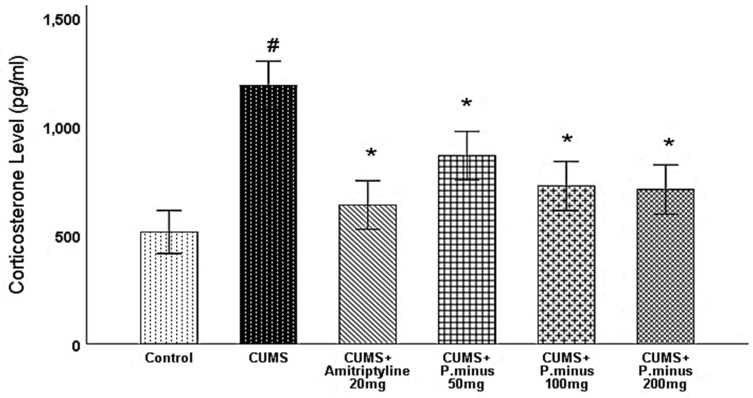
Effects of *P. minus* aqueous extract on serum corticosterone level (pg/mL) in CUMS induced depressive mice. **#** (*p <* 0.05), vs. Control group; * (*p <* 0.05), vs. CUMS group, (*n* = 6).

As described in Figure 7, CUMS treated group has significantly enhanced the serum corticosterone level with a comparison of Control. An enhanced corticosterone level was significantly decreased in all treatment groups. Reduction of corticosterone level in CUMS + Amitriptyline 20 mg was more than *P. minus* treated groups, whereas CUMS + *P. minus* 50 mg treated group showed a minimum decrease as compared to other groups. The reduction pattern in CUMS+ *P. minus* 100 mg and CUMS + *P. minus* 200 mg was quite similar.

#### 3.6.2. Effects of *P. minus* Aqueous Extract on Brain-Derived Neurotrophic Factor (BDNF) Level

**Figure 8:**
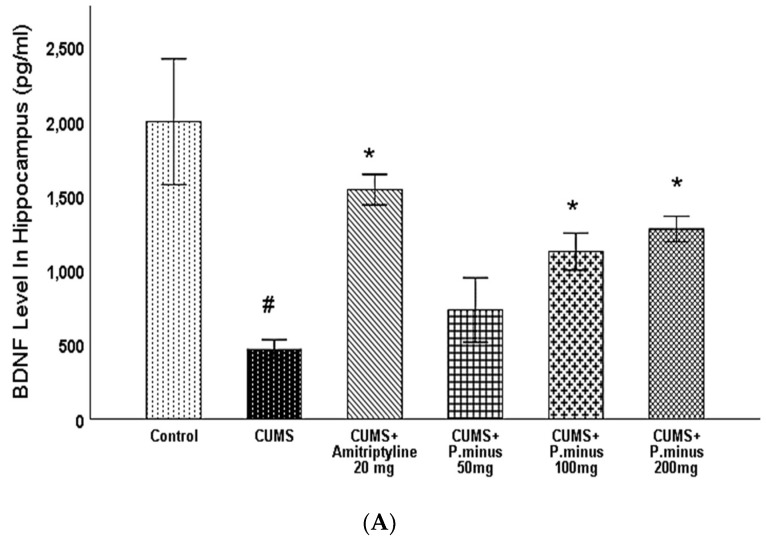

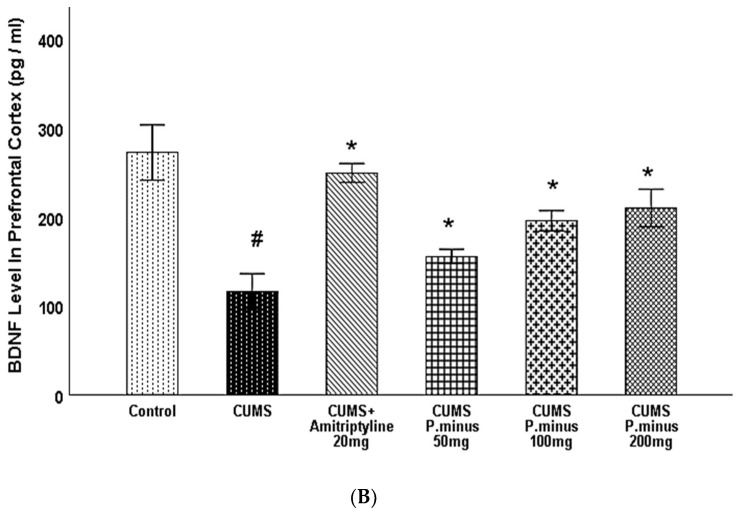
Effects of *P. minus* aqueous extract on BDNF level in CUMS induced depressive mice. (**A**) BDNF level in Hippocampus(pg/mL) (**B**) BDNF level in Prefrontal cortex (pg/mL). # (*p <* 0.05), vs. Control group; * (*p <* 0.05), vs. CUMS group, (*n* = 6).

Figure 8A, B presents the results of BDNF levels influenced by treatment and CUMS in the hippocampus and prefrontal cortex. Variations of BDNF levels in the hippocampus are expressed, which demonstrates that CUMS group had significantly reduced the BDNF level in the hippocampus. This reduction was inverted by CUMS + Amitriptyline+ 20 mg, *P. minus* 200 mg and100 mg treated groups significantly, but CUMS + *P. minus* 50 mg treated group did not show any significant variations in hippocampal BDNF level. In Figure 8B BDNF level variations in the prefrontal cortex are shown, according to these results CUMS also decreased the BDNF level in the prefrontal cortex but this effect was reversed by all treatment groups significantly. The CUMS + Amitriptyline 20 mg showed more improvement in BDNF levels in the hippocampus and prefrontal cortex as compared to *P. minus* treated groups

#### 3.6.3. Effects of *P. minus* Aqueous Extract on Monoamine Oxidase -A (MAO-A)

**Figure 9:**
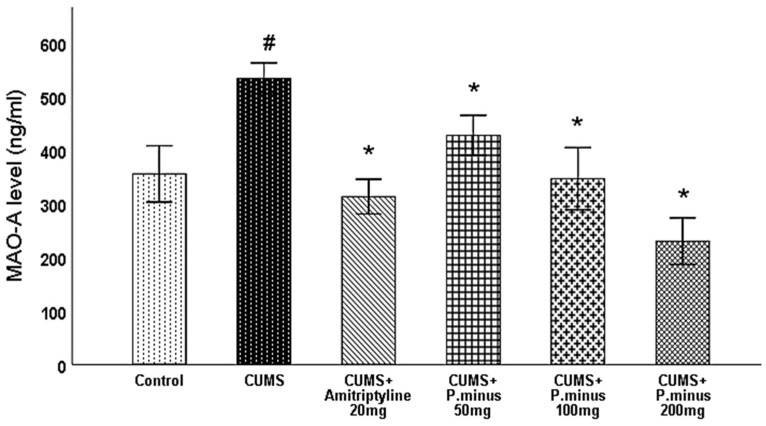
Effects of *P. minus* aqueous extract on MAO-A level (ng/mL) in CUMS induced depressive mice. **#** (*p <* 0.05), vs. Control group; * (*p <* 0.05), vs. CUMS group, (*n* = 6).

MAO-A level was measured from the hippocampus of the brain. Figure 9 shows a significantly increased level of MAO-A in CUMS group as compared to the control group which was significantly reversed in all treatment groups whereas more MAO-A inhibition was observed with administration of CUMS + *P. minus* 200 mg than in other treatment groups.

#### 3.6.4. Effects of *P. minus* Aqueous Extract on Serotonin (5-HT) Level

**Figure 10:**
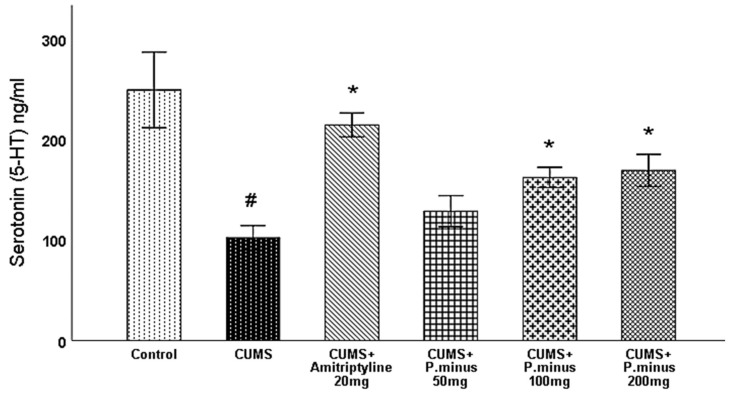
Effects of *P. minus* aqueous extract on Serotonin (5-HT) level(ng/mL) in CUMS induced depressive mice. **#** (*p <* 0.05), vs. Control group; * (*p <* 0.05), vs. CUMS group, (*n* = 6).

In this section serotonin results measured from the hippocampus are discussed, as per Figure 10 serotonin level was significantly reduced in CUMS group as compared to the control. CUMS+ amitriptyline 20 mg, CUMS +*P. minus* 100 mg and *P. minus,* 200 mg groups showed a significant increase in serotonin (5-HT) level, whereas CUMS + *P. minus* 50 mg did not show a significant difference in serotonin level in comparison to CUMS group. These results indicate that administration with higher doses of *P. minus* can enhance the level of serotonin in the hippocampus.

#### 3.6.5. Effects of *P. minus* Aqueous Extract on Norepinephrine (NE) Level

**Figure 11:**
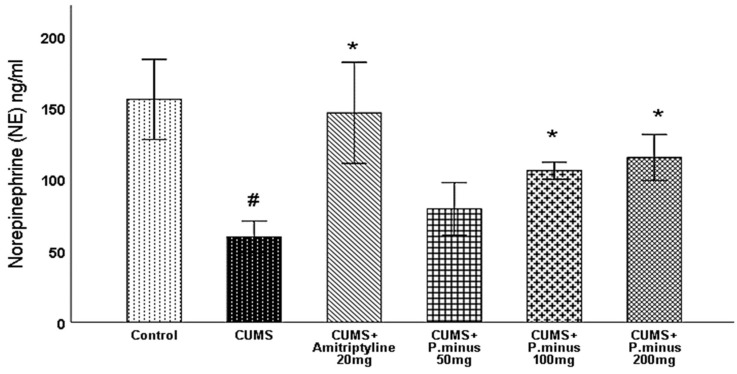
Effects of *P. minus* aqueous extract on Norepinephrine (NE) level(ng/mL) in CUMS induced depressive mice. **#** (*p <* 0.05), vs. Control group; * (*p <* 0.05), vs. CUMS group, (*n* = 6).

Norepinephrine level was also observed in the hippocampus part of the brain, According to Figure 11, CUMS + Amitriptyline 20 mg, *P. minus* 100 and 200 mg treated groups showed a significant increase in norepinephrine level in comparison to CUMS group. While CUMS group decreases the norepinephrine level significantly as compared to the control group. In addition, CUMS + *P. minus* 50 mg has not shown any significant difference in comparison to CUMS group

## 4. Discussion

BioKesum^®^ extract is registered under the National Pharmaceutical Control Bureau (NPCB) (Reg# MAL14015033T). BioKesum^®^ contains bioactive compounds including quercetin-3-glucuronide (not less than 0.45%), and quercitrin (not less than 0.15%), These are the most abundant compounds in the total phenolic extract of *P. minus* aqueous extract of leaf part [21]. 

Our primary finding of this research work showed that administration with *P. minus* aqueous extract (BioKesum^®^) has a significant role in improvement on CUMS induced cognition deficit and depression with maximum doses (*P. minus* 100 mg, *P. minus* 200 mg). A previously done work on the effects of *P. minus* extract on scopolamine-induced cognition deficit supports the results (Figure 5) of this current study. But the cognition deficit induction method was different in both studies. That work also suggests that this property of *P. minus* aqueous extract is due to the presence of quercitrin and quercetin 3 glucuronide (Q3G) like flavonoids. Q3G is an abundant compound in this extract, which is a derivative of quercetin [20]. According to previously done studies, it is proved that quercetin 3 glucuronide and quercitrin cross the blood-brain barrier and can be absorbed from the intestine and further distributed to the central nervous system [30,31]. Recent research explained that *P. minus* extract can enhance mood and BDNF levels in a human trial [21], through results (Figure 8) of the current study we support their findings as shown which indicates that *P. minus* extract administration significantly enhanced the BDNF level in CUMS induced depressive mice. 

Despite all these findings and studies, there was a gap in research regarding its antidepressant effects because no proper mechanisms were found in all previously done work regarding its beneficial activities. So, the current study demonstrates some potential regarding anti-depressant and cognitive enhancing properties of *P. minus* aqueous extract. 

This research showed that *P. minus* has the potential to reverse the CUMS induced behavioral disturbances significantly by using the method of sucrose preference test, Barnes maze assay and forced swimming test with higher doses of *P. minus* 100 mg and *P. minus* 200 mg mostly. Our findings regarding antidepressant effect are strongly supported by previously done work on individual flavonoids which are present in *P. minus* extract, Quercitrin (quercetin 3-rhamnoside) alleviated depression-like behaviors with a maximum dose (10 mg/kg) among depressive mice in a previous study [32]. Whereas for Q3 G, a researcher suggested that it has a potential role in the antidepressant effect of *Hypericum perforatum* (St. John’s wort) through forced swimming test in mice, it can reduce the immobility time significantly which is similar to our result (Figure 6) of immobility time [33]. In the case of anxiety behavior, *P.minus* treated groups showed a decrease in the anxiety level through open field test in comparison to CUMS but it was not significantly (Figure 4), The same pattern was also observed in another study in which quercetin 3 glucuronide showed non-significance improvement on anxiety as a part of Hypericum perforatum (St. John’s wort) with 0.6 mg/kg dose through open field test [34], but it has already reversed the immobility time in forced swimming test by the same researcher in another study [33]. It is suggested that might be significant anxiolytic effect can be achieved by increasing the duration of time. *P. minus* aqueous extract also significantly inhibit the MAO-A level in CUMS induced depressive mice which clearly expressed that *P. minus* extract may be proven as an MAO-A inhibitor by its anti-depressant mechanism of action as shown in Figure 9. As MAO-A inhibition is directly involved to increase the level of the monoamine because it is involved in the degradation of serotonin and norepinephrine, its inhibition is directly proportional to enhancing the level of the monoamine, in our experiment *P. minus* aqueous extract aqueous has significantly increased the serotonin and norepinephrine with maximum doses (*P. minus* 100 mg and *P. minus* 200 mg) only (Figure 10 and Figure 11). These results are supported by some previously done studies, in which quercetin 3 glucuronide (Q3G) was a part of *Apocynum venetum* leaves extract, that extract increased the level of serotonin and norepinephrine level in the depression model [35]. If we discuss furthermore another hypothesis of depression model HPA axis, we can observe that Corticosterone level is the main factor in this theory which indicates that increased cortisol or corticosterone level is the major cause of depression, our results showed that *P. minus* aqueous extract has reduced the corticosterone level significantly as compare to chronic ultra-mild stress (CUMS) induced depressive mice group (Figure 7). It is recommended that a future study should be done on each constituent of this extract especially on Q3G because it has very less reported literature regarding anti-depressant effects as an individual constituent, it needs a separate full comprehensive research study which will describe its doses and will demonstrate its various beneficial and side effects along with the dose-response relationship. Based on all current findings, it is suggested that quercetin 3-glucuronide and quercitrin (quercetin 3-rhamnoside) are may be responsible for the anti-depressant-like effects of *P. minus* aqueous extract, quercetin-3-glucuronide is the most abundant constituent (0.45%) in *P. minus* aqueous extract and other studies showed that 0.6 mg/kg dose of this constituent had significant antidepressant potential which was near to concentration used in current research even *P. minus* 200 mg/kg (maximum dose group) had more concentration of Q3G as compare to that extract which was used in previous studies [33], whereas quercitrin showed a significant increase in monoamine level and antidepressant potential with 10 mg/kg only in previous studies, even nothing was significant with 5 mg/kg dose in one of a previous study [32,36]. Based on previous evidence, the concentration of quercitrin in the present extract (*P. minus* aqueous extract) was not sufficient for significant anti-depressant potential. 

In the case of *P. minus* aqueous extract (BioKesum), it is strongly recommended to develop its planned and prescribed use for improving the memory and depression signs based on its beneficial mechanisms of action, observed in current research work.

## 5. Conclusions

It is concluded that *P. minus* aqueous extract has an anti-depressant-like effect on behavior, it shows an improved spatial memory, reduced immobility, decreased corticosterone level, elevated serotonin, norepinephrine and BDNF level along with a significant reduction of MAO-A level compared to CUMS induced depressive mice model.

## Figures and Tables

**Table 1:** Chronic ultra-mild stress weekly protocol.

Days	First Stress	Duration	Second Stress	Duration
1	Crowded housing	6 h	Cage tilt	8 h
2	Restricted access to Food and water	8 h	Continuous light	Over the night
3	Forced swimming	20 min	Tail pinch	2 min for each
4	Restraint stress	2 h	Foreign object in	Over the night
5	Cage tilt	8 h	Continuous light	Over the night
6	Restricted access to food	8 h	Crowded Housing	6 h
7	Forced swimming	20 min	Wet bedding	Over the night

## Data Availability

The data presented in this study are available on request from the corresponding author.
